# Immunogenomic Landscape and Immune-Related Gene-Based Prognostic Signature in Asian Gastric Cancer

**DOI:** 10.3389/fonc.2021.750768

**Published:** 2021-11-05

**Authors:** Chenchen Mao, Liangliang Ma, Yingpeng Huang, Xinxin Yang, He Huang, Wentao Cai, Andriamifehimanjaka Sitrakiniaina, Ruihong Gu, Xiangyang Xue, Xian Shen

**Affiliations:** ^1^Department of Gastrointestinal Surgery, The Second Affiliated Hospital of Wenzhou Medical University, Wenzhou, China; ^2^Department of Vascular Surgery, The Second Affiliated Hospital of Wenzhou Medical University, Wenzhou, China; ^3^Department of General Surgery, The Second Affiliated Hospital, Wenzhou Medical University, Wenzhou, China; ^4^Department of Microbiology and Immunology, School of Basic Medical Sciences, Institute of Molecular Virology and Immunology, Wenzhou Medical University, Wenzhou, China

**Keywords:** gastric cancer, Asian cancer, immunomodulatory gene, immunotherapy, prognostic gene signature

## Abstract

**Background:**

Asians have the highest incidence of gastric cancer (GC), and the prognosis of Asian GC is poor. Furthermore, the therapeutics for Asian GC is limited because of genetic heterogeneity and screening difficulty at the early stage. This study aimed to develop an immune-related gene (IRG)-based prognostic signature and to explore prognosis-related regulatory mechanism and therapeutic target for Asian GC.

**Methods:**

To elucidate the prognostic value of IRGs in Asian GC, a comprehensive analysis of IRG expression profiles and overall survival times in 364 Asian GC patients from the Asian Cancer Research Group (ACRG) and The Cancer Genome Atlas (TCGA) databases was performed, and a novel prognostic index was established. To further explore regulatory prognosis mechanisms and therapeutic targets, a tumor immunogenomic landscape analysis, including stromal and immune subcomponents, cell types, panimmune gene sets, and immunomodulatory genes, was performed.

**Result:**

Our analysis allowed the creation of an optimal risk assessment model, the Asian-specific IRG-based prognostic index (ASIRGPI), which showed a high accuracy in predicting survival in Asian GC. We also developed an ASIRGPI-based nomogram to predict the 3- and 5-year overall survival (OS) of Asian GC patients. The impact of the ASIRGPI on the worse prognosis of Asian GC was possibly related to the stromal component remodeling. Specifically, TGFβ gene sets were significantly associated with the ASIRGPI and worse prognosis. Immunomodulatory gene analysis further revealed that TGFβ1 and EDNRB may be the novel potential therapeutic targets for Asian GC.

**Conclusions:**

As a tumor microenvironment-relevant gene set-based prognostic signature, the ASIRGPI model provides an effective approach for evaluating the prognosis of Asian GC and may even prolong OS by enabling the selection of individualized therapy with the novel targets.

## Introduction

Gastric cancer (GC) was the fourth most common malignant tumor and the third leading cause of cancer mortality in 2019 according to global cancer statistics (1, 2). The morbidity of GC varies greatly across regions, with nearly 60% of GC occurring in East Asia (3), which has the highest cancer-related mortality for GC worldwide. Additionally, studies have also shown that Asian Americans have a higher GC incidence than non-Hispanic whites (4–6), which indicates that Asian GC (AGC) is associated not only with lifestyle and culture but also with genetics. Previous research (5–7) reported that the prognosis of AGC is better as it is often diagnosed at earlier tumor stages, at a more distal anatomic site, and at a younger age and receives more aggressive treatment. Considering that the prognosis of GCs depends not only on tumor stage but also on heterogeneous and epigenetic molecular features (8, 9), the differences in GC survival patterns and the possible causes of survival disparities among different ethnic groups have not yet been clarified. Thus, elucidating the mechanisms of AGC will offer new insights for prognosis prediction and treatment of GC.

Over the past decade, immunotherapy, especially immune check inhibitors (ICIs), has become a promising treatment strategy (10, 11). Chen et al. demonstrated that anti-PD1, anti-PD-L1, and anti-CTLA-4 ICIs could improve some survival endpoints in advanced GCs (12), indicating that immunotherapeutic approaches have promising prospects for long-term and durable remission of GCs. However, the effect of immunotherapy for GC is limited, and there are great individual differences in GC immunotherapy. Additionally, the tumor microenvironment (TME), including the extracellular matrix (ECM), stromal cells, and immunoinfiltrating cells, was found to play significant roles in the progression, metastasis, and therapeutic response of a variety of tumors (13, 14). Li et al. developed a TME-based risk score as an independent prognostic factor for GC (15). Studies have also shown that a high M2 macrophage level is related to the status of peritoneal dissemination, angiogenesis, immune evasion, and poor prognosis in GCs (16, 17). Thus, TME-related gene set-based prognostic signatures might be immunotherapic signs in GCs.

In this study, we systematically characterized the immunogenomic landscape and immune-related gene (IRG) signatures of Asian and white GC by investigating the TCGA-STAD and ACRG transcriptional profiles. We further determined the clinical role of immune genes as tools for classifying the prognoses of AGC patients and developed and validated an individualized Asian-specific IRG-based prognostic index (ASIRGPI). Furthermore, the relationship between ASIRGPI and the TME was analyzed to explore ASIRGPI-related survival mechanisms and therapeutic targets.

## Materials and Methods

### Ethical Information and Study Cohorts

This study was approved by the local Research Ethics Board of The Wenzhou Medical University Second Affiliated Hospital, and informed patient consent was waived. All procedures performed in studies involving human participants were in accordance with the 1964 Helsinki declaration and its later amendments.

Gene sequencing data and the corresponding clinical data for stomach adenocarcinoma (STAD) and adjacent normal tissue samples were downloaded from The Cancer Genome Atlas database (TCGA; https://portal.gdc.cancer.gov). In addition, the raw GSE62254/ACRG (Asian Cancer Research Group) data, containing 300 AGC patients, including survival information, were downloaded from the Gene Expression Omnibus database (GEO: https://www.ncbi.nlm.nih.gov/geo/). Z-score normalization was performed for all gene sequencing data.

### Prognostic Model Establishment

Differentially expressed genes (DEGs) between tumor and adjacent normal tissues were determined with the filtering condition of log2 |fold change | > 1 and a false discovery rate (FDR) < 0.05, using the limma package in R software 3.6.3. The IRG list, which has been validated to be involved in immunity, was downloaded from ImmPort, a platform that provides accurate and timely immunological data. Differentially expressed IRGs were thus extracted from the DEGs.

Patients who have an overall survival (OS) of <90 days as well as missing survival information were excluded from further study. Thus, a total of 364 patients were finally enrolled. Univariate Cox and further LASSO regression analysis was used to establish the prognostic model. The survival receiver operating characteristic (ROC) curve was performed to evaluate the performance of the ASIRGPI and to determine the cutoff for classifying AGC patients as low- or high-risk. Survival analysis associated with the prognostic model was carried out *via* Kaplan–Meier analysis.

Furthermore, multivariate Cox regression analyses were used to evaluate whether the prognostic model could independently predict the prognosis of AGCs. A nomogram was thus formulated using the coefficients of the multivariable Cox regression model *via* the rms package in R. Calibration curves were assessed graphically by plotting the observed rates against the nomogram-predicted probabilities.

### TME Characterization

**Figure 1:**
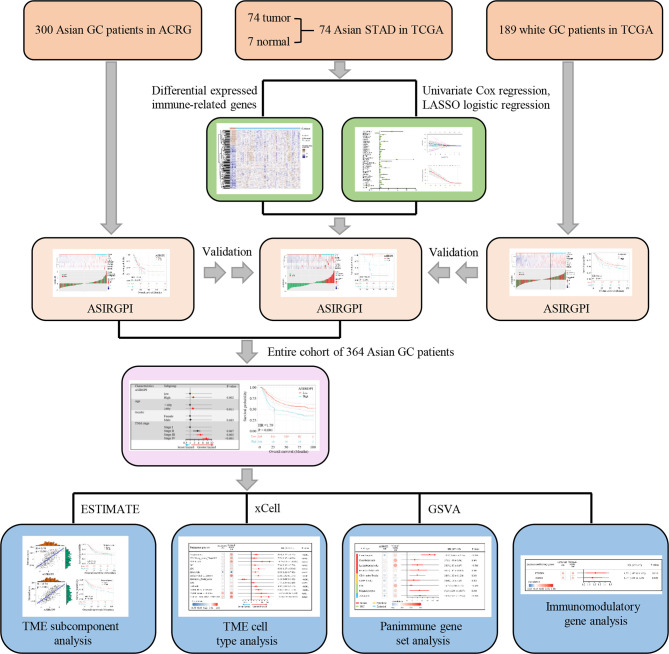
Study flowchart.

ESTIMATE, which uses gene expression signatures to infer stromal and immune cell fractions to determine stromal and immune scores, was performed to analyze the TME subcomponent, while xCELL, which also uses gene expression to infer the proportions of 64 tumor-infiltrating immune cell (TIIC) and stromal cell types, was further used to analyze the TME cell type. Additionally, gene set variation analysis (GSVA) was performed to estimate the enrichment scores of 110 immunoregulation-related pathways in AGC while the immunomodulatory gene was estimated *via* analyzing 78 immunomodulatory genes summarized by the TCGA immune response working group. A detailed flowchart is shown in **Figure 1**.

### Immunohistochemistry Detection

Tissue microarrays from 154 local AGC patients (35 tissue pairs and 119 GC tissue) were constructed according to our previous research (18). IHC was performed in the Pathology Laboratory of The Second Affiliated Hospital of Wenzhou Medical University, using rabbit anti-TGFB1 monoclonal antibody (Abcam, cat. no. ab27969) at a dilution of 1:250. The results were initially determined by two pathology experts and were accepted if a third expert also confirmed the result. Otherwise, the data were reviewed by all three experts and discussed until a consensus was reached. Finally, a computer-automated method was conducted (Image-Pro Plus 6.0; Media Cybernetics, Inc.) and the expression level was represented as numbers of positive cells per square millimeter (positive cells number/total area).

### Statistical Analysis

All statistical analyses are carried out using R (version 3.6.3) and SPSS 22.0. Pearson correlation analysis was used to determine the correlation, and survival analysis was performed using the log-rank test. p < 0.05 was considered statistically different.

## Results

### Landscape of IRGs and TME in Asian GC

**Figure 2:**
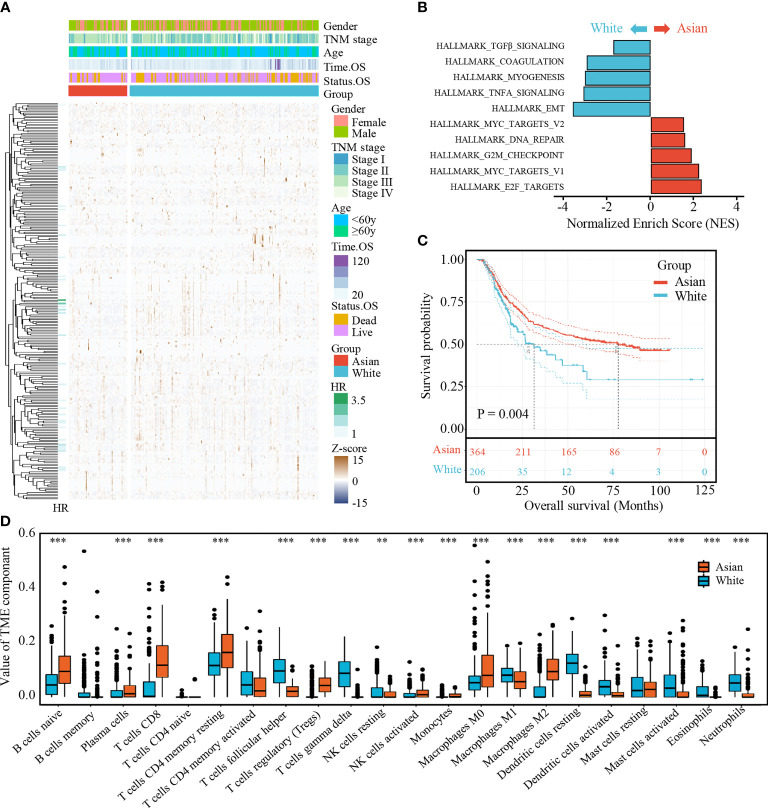
**(A)** Expression profile of 250 IRGs in TCGA-STAD Asian cohort and TCGA-STAD white cohort. **(B)** Gene set enrichment analysis (GSEA) of hallmark gene sets. **(C)** Kaplan-Meier curves for overall survival of Asian cohort and white cohort. The number of patients in Asian cohort and white cohort are n = 364 and n = 206, respectively. The statistical difference between three survival curves was tested by log-rank test. **(D)** The boxplot of TME component in two cohorts. Within each group, the scattered dots represent TME fraction expression values. The thick line represents the median value. The bottom and top of the boxes are the 25th and 75th percentiles (interquartile range). **P < 0.01 and ***P < 0.001.

To identify Asian-specific IRGs, we first selected all Asian tissues (74 tumor and 7 adjacent normal tissues) in the TCGA-STAD cohort and the differential expression patterns of 2,498 IRGs in AGC tissues were further analyzed to form an expression profile to analyze differentially expressed IRGs. We determined 685 DEGs, including 515 upregulated and 130 downregulated genes between GC and adjacent normal samples, using the limma package with cutoffs |log fold change| > 1 and FDR < 0.05. After removing genes not detected in the ACRG dataset, 183 upregulated and 67 downregulated IRGs were finally identified (**Supplementary Figures S1A, B**). The expression profiles of the 250 IRGs in the TCGA-STAD Asian cohort and TCGA-STAD white cohort are shown in **Figure 2A**. Further, gene set enrichment analysis (GSEA) revealed that TGFβ signaling, coagulation, myogenesis, TNFA signaling, and epithelial–mesenchymal transition (EMT) were enriched in the TCGA-STAD white cohort, while MYC targets V2, DNA repair, G2M checkpoint, MYC targets V1, and E2F targets were enriched in the TCGA-STAD Asian cohort (**Figure 2B** and **Supplementary Figure S2**).

To further explore the survival differences between the different ethnicities, a Kaplan–Meier survival curve was performed. After excluding patients with an OS of <90 days and those without survival information, we found that white GC patients (TCGA-STAD white cohort) correlated with poor prognosis compared with AGC patients (ACRG cohort and TCGA-STAD Asian cohort; p = 0.004, **Figure 2C**).

Meanwhile, statistical differences of infiltrating TME cells between the Asian and white GC patients were investigated; we found that the fractions of B cells naïve, plasma cells, T cells CD8, T cells CD4 memory resting, T cells regulatory, NK cells resting, NK cells activated, monocytes, macrophages M0, and macrophages M2 were significantly increased in AGC, while T cells follicular helper, T cells gamma delta, macrophages M1, dendritic cells resting, dendritic cells activated, mast cells activated, eosinophils, and neutrophils were significantly decreased (**Figure 2D**).

### Generation and Validation of ASIRGPI in TCGA and ACRG Cohorts

**Figure 3:**
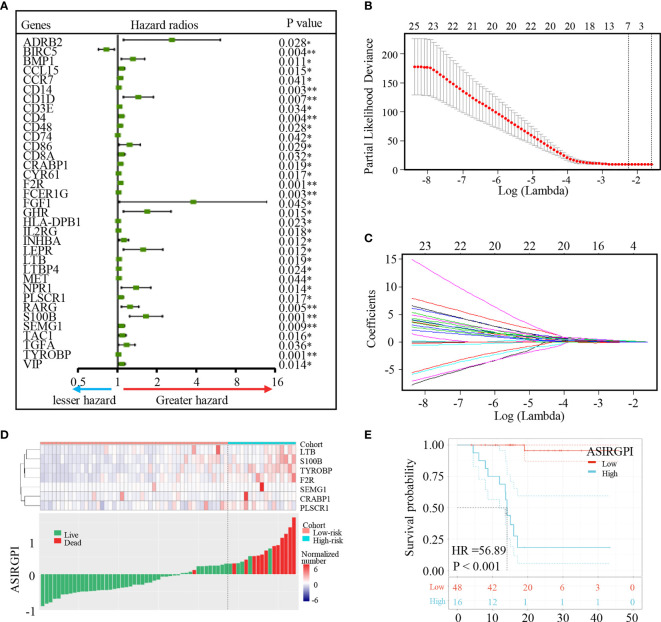
Identification of Asian-specific survival-associated IRGs and generation of the ASIRGPI in the TCGA-STAD Asian cohort. **(A)** Forest plot of hazard ratios of prognostically relevant immune genes, revealing a prognostic value in AGC. *P < 0.05, **P < 0.01. **(B, C)** Least absolute1 shrinkage and selection operator (LASSO) regression was performed, calculating the minimum criteria. **(D)** Patients were divided into high-risk and low-risk groups with ASIRGPI score = 0.308585 utilized as the cutoff value. The relationships between the expression of seven prognostic genes (upper) and risk score distribution with survival status (bottom) in the TCGA-STAD Asian cohorts. **(E)** Kaplan–Meier curves for the high (n = 16) and low (n = 48) risk score patient groups in the TCGA-STAD Asian cohort. Log-rank test, p < 0.001.

To further explore the clinical significance of differentially expressed IRGs, univariate Cox regression analysis was performed and 35 IRGs were found to be survival-associated using the 64 AGC samples and clinical information for the TCGA-STAD cohort (**Figure 3A**). LASSO regression analysis further reduced 35 potential IRGs to 7, which had non-zero coefficients in the regression model (**Figures 3B, C**). An Asian-specific IRG-based prognostic index was calculated for each patient using the following formula: ASIRGPI = (0.0146 * expression of CRABP1) + (0.2543 * expression of F2R) + (0.0413 * expression of LTB) + (0.1680 * expression of PLSCR1) + (0.2501 * expression of S100B) + (0.0979 * expression of SEMG1) + (0.1692 * expression of TYROBP).

ROC analysis was carried out to assess the ASIRGPI. The high area under the curve (AUC of 0.903) confirmed the high prognostic performance of the ASIRGPI in survival surveillance. Additionally, the maximal Youden index value for an ROC curve was determined as the optimal cutoff point (0.3086; **Supplementary Figure S3**). AGC patients in the TCGA were thus assigned into low- and high-risk cohorts. High-risk patients (16, 25.0%) had shorter OS (HR = 56.89, p < 0.001) than low-risk patients (48, 75.0%) among the 64 Asian patients with GC (**Figure 3E**). The expression of the seven prognostic genes and the relationship between ASIRGPI distribution and the survival status are shown in **Figures 3D, E**.

**Figure 4:**
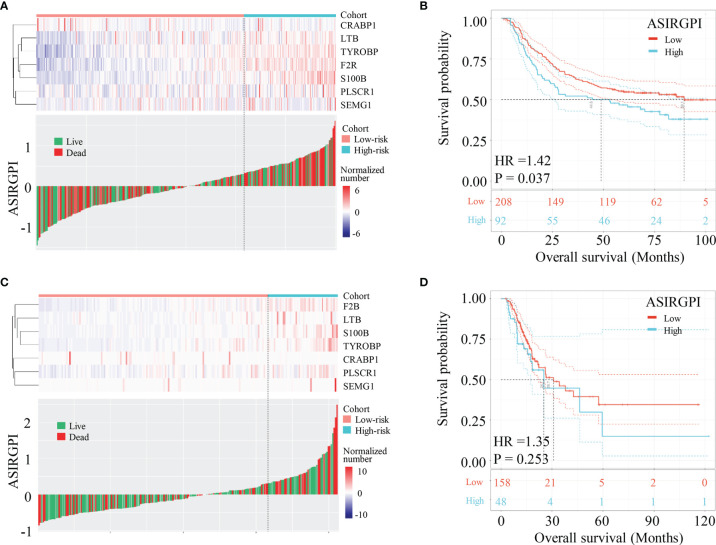
Validation of ASIRGPI in the TCGA-STAD white cohort and ACRG cohorts. **(A, B)** The relationships between the expression of seven prognostic genes (upper) and risk score distribution with survival status (bottom) in the ACRG. Kaplan–Meier curves for the high (n = 92) and low (n = 208) risk score patient groups in the ACRG cohort. Log-rank test, p = 0.037. **(C, D)** The relationships between the expression of seven prognostic genes (upper) and risk score distribution with survival status (bottom) in the TCGA-STAD white cohorts. Kaplan–Meier curves for the high (n = 46) and low (n = 143) risk score patient groups in the TCGA-STAD white cohort. Log-rank test, p = 0.630.

To further validate our ASIRGPI, its performance was assessed in the ACRG cohort, which consisted of 300 AGC patients. Consistently, high-risk patients (92, 30.7%) had a worse prognosis than low-risk patients (208, 69.3%) in the ACRG validation cohort (HR =1.42, p = 0.037; **Figures 4A, B**). Interestingly, for the white GCs of the TCGA-STAD cohort, ASIRGPI categorized 143 (75.7%) and 46 (24.3%) patients into the low-risk and high-risk groups, respectively. However, no OS difference was found (HR =1.14, p = 0.630; **Figures 4C, D**) between the two groups.

### Clinical Correlation Analysis and Construction of ASIRGPI-Based Nomogram

**Figure 5:**
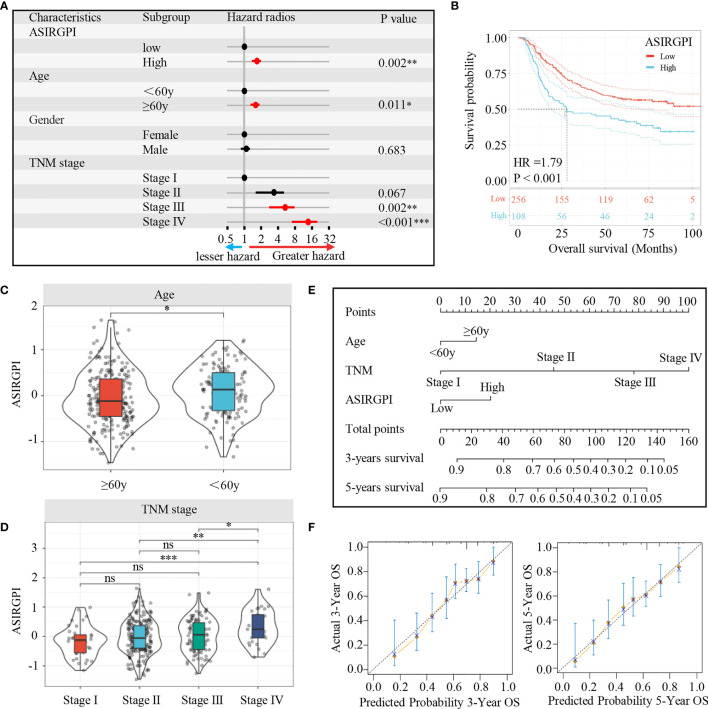
**(A)** Multivariate Cox analysis evaluating independently predictive ability of the risk score and other clinical risk factors for OS. The square data markers indicate estimated hazard ratios. The error bars represent 95% CIs. **(B)** Kaplan–Meier curves for the high (n = 108) and low (n = 256) ASIRGPI patient groups in the Asian GCs. Log-rank test, P < 0.001. **(C, D)** The relationship between ASIRGPI with clinical and demographic characteristics. **(E)** Development of a nomogram based on the ASIRGPI to predict the 3-year and 5-year overall survival for AGC patients. **(F)** Calibration plot of the nomogram for predicting the probability of OS at 3, and 5 years in Entire cohort of 364 AGC patients. ns, no significance, *P < 0.05, **P < 0.01 and ***P < 0.001.

The clinical characteristics of the patients in the TCGA and ACRG cohorts are depicted in **Table 1**. To explore the prognostic value of ASIRGPI, multivariate Cox regression analyses were conducted for all 364 AGC patients. We found that the ASIRGPI, age, and TNM stage serve as independent predictors of AGC patient survival outcomes (**Figure 5A**). Similarly, high-risk patients (88, 29.7%) had shorter OS (HR = 1.79, p < 0.001) than low-risk patients (256, 70.3%; **Figure 5B**).

**Table 1:** Clinical characteristics of the GC patients used in this study.

	TCGA Asian cohort	TCGA white cohort	ACRG Asian cohort
No. of patients	64	206	300
Age			
<60 (%)	25 (39.06%)	62 (30.10%)	106 (35.33%)
≥60 (%)	39 (60.94%)	144 (69.90%)	194 (64.67%)
Gender (%)			
Female	39 (60.94%)	75 (36.41%)	101 (33.67%)
Male	25 (39.06%)	131 (63.59%)	199 (66.33%)
Grade (%)			
Grade 1	3 (4.69%)	5 (2.43%)	NA
Grade 2	20 (31.25%)	72 (34.95%)	NA
Grade 3	40 (62.50%)	124 (60.19%)	NA
Grade X	1 (1.56%)	5 (2.43%)	NA
Stage (%)			
I	5 (7.81%)	12 (5.83%)	31 (10.33%)
II	41 (64.06%)	37 (17.96%)	97 (32.33%)
III	16 (25.00%)	66 (32.04%)	95 (31.67%)
IV	2 (3.13%)	4 (19.42%)	77 (25.67%)
Lauren			
Intestinal	NA	NA	146 (48.67%)
Diffuse/mix	NA	NA	152 (50.67%)
Location			
Antrum	NA	NA	150 (50.00%)
Cardia	NA	NA	30 (10.00%)
Body	NA	NA	115 (38.33%)
Whole	NA	NA	4 (1.33%)

To further elaborate the clinical significance of the ASIRGPI, the relationship between the ASIRGPI and clinical and demographic characteristics, including age and TNM stage (T stage, lymphatic invasion, and distant metastasis), according to the International Union against Cancer was analyzed. We found that the ASIRGPI was positively correlated with both age and TNM stage (**Figures 5C, D**).

Finally, to provide a more intuitive clinical application tool, a nomogram was integrated with the ASIRGPI; age and TNM stage were constructed based on multivariate Cox analysis (**Figure 5E**). Calibration plots showed that the nomogram could predict the probability of 3- and 5-year OS well (**Figure 5F**).

### Association of TME Subcomponents and Cell Types With ASIRGPI and AGC Patient Outcomes

**Figure 6:**
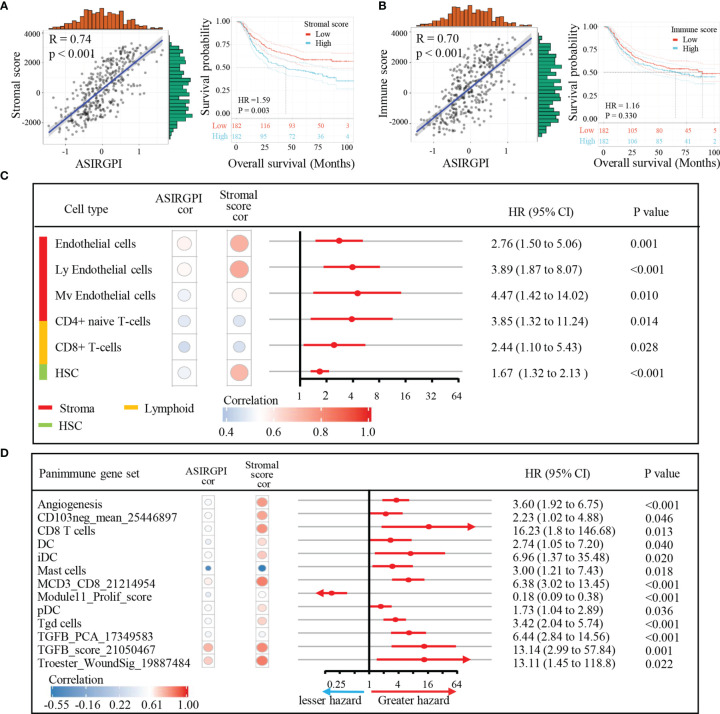
Association of TME subcomponents and TME cell types with ASIRGPI and outcome in AGC patients. **(A, B)** Association of TME subcomponents with risk score and outcome in the entire 364 AGC cohort. **(A)** Scatter plots depicting the positive correlation between stromal score and ASIRGPI in AGC patients. Pearson’s correlation coefficient is shown in the graphs (p < 0.001). Kaplan–Meier curves for overall survival of 364 AGC patients according to stromal score. Log-rank test, p = 0.003. **(B)** Scatter plots depicting the positive correlation between immune score and ASIRGPI. Pearson’s correlation coefficient is shown in the graphs (p < 0.001). Kaplan–Meier curves for overall survival of 364 AGC patients according to immune score. Log-rank test, p = 0.330. **(C)** TME cell type analysis, xCell, a method that uses gene expression signatures to infer the proportions of 64 immune and stromal cell types in samples, was utilized to determine the enrichment score of each cell type *via* ssGSEA. Among the 64 TME cell types, those significantly related to overall survival (log-rank test, p < 0.05) and risk score (Pearson’s correlation test, |r| ≥ 0.40, p < 0.05) are listed. The circular data markers indicate estimated hazard ratios. The error bars represent 95% CIs. Pearson’s correlation coefficients between nine TME cell types and stromal scores are also shown (p < 0.05). **(D)** Panimmune gene set analysis, gene set variation analysis (GSVA) was used to estimate the enrichment scores of 110 immunoregulation-related pathways in AGC samples. Among the 110 panimmune gene sets, those significantly related to overall survival (log-rank test, p < 0.05) and risk score (Pearson’s correlation test, |r| ≥ 0.40, p < 0.05) are listed. The circular data markers indicate estimated hazard ratios. The error bars represent 95% CIs. Pearson’s correlation coefficients between 13 panimmune gene sets and stromal scores are also shown (p < 0.05).

To explore potential ASIRGPI-related survival mechanisms, ESTIMATE was performed and stromal/immune scores were inferred *via* ssGSEA for the entire cohort. Pearson’s correlation analysis showed that both the stromal and immune scores were positively correlated with ASIRGPI [r = 0.74, p < 0.001 (**Figure 6A**) and r = 0.70, p < 0.001 (**Figure 6B**), respectively]. Additionally, using the median score as the cutoff values, survival analysis showed that high-stromal-scored AGC patients had a worse OS than low-stromal-scored patients (HR = 1.59, p = 0.003; **Figure 6A**), but no OS difference was observed between low- and high-immune-scored patients (HR = 1.16, p = 0.330; **Figure 6B**). Furthermore, TME cell-type analysis was performed and 64 TME cell types were inferred. Among these, nine cell types (**Figure 6C**) were both significantly related to overall survival (log-rank test, p < 0.05) and the ASIRGPI (|r| ≥ 0.40, p < 0.05). Remarkably, four stromal cell types with the largest proportions as well as five other cell types, including lymphoid, hematopoietic stem, and epithelial cells, were all positively associated with poor prognosis and ASIRGPI.

### TGFB1 Validation as the Therapeutic Target in AGC Patients

Next, to explore potential ASIRGPI-associated therapeutic targets, GSVA was performed to estimate the enrichment scores of the 110 immunoregulation related pathways. We found 13 panimmune gene sets to be significantly associated with overall survival (P < 0.05) and with ASIRGPI (|r| ≥ 0.40, P < 0.05); 11 were positively associated with worse outcomes and ASIRGPI risk scores (**Figure 6D**). The remaining two genes were positively associated with worse outcomes and negatively associated with ASIRGPI risk scores, while the Module11_Prolif_score was the opposite. Interestingly, TGFβ-related gene sets (TGFB_PCA_17349583 and TGFB_score_21050467), which are closely associated with the remodeling of stromal components in the TME, were highlighted during this screening.

**Figure 7:**
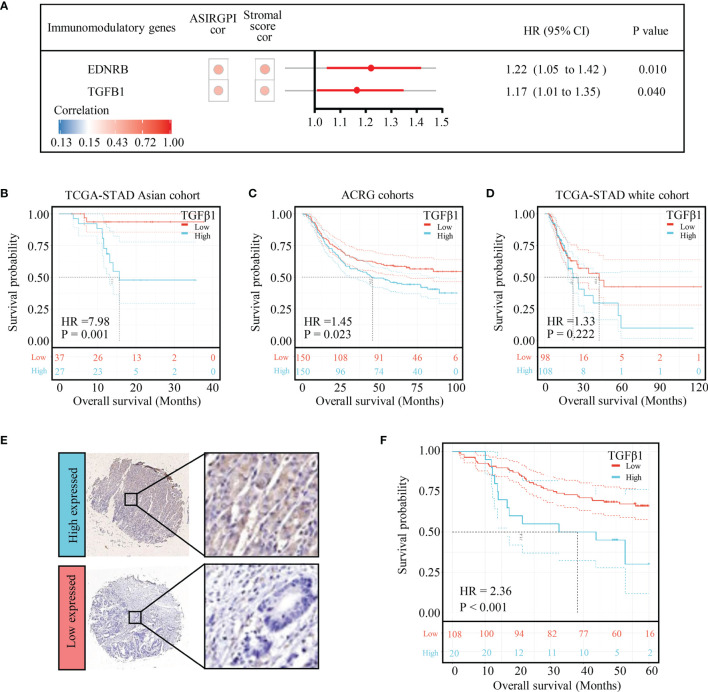
TGFB1 was validated to be the therapeutic target in AGC patients. **(A)** Among the 29 immunomodulatory genes detected in both TCGA and ACRG databases, only EDNRB and TGFB1 were related to risk score (Pearson’s correlation test, |r| ≥ 0.40, p < 0.05) as well as overall survival (log-rank test p-value < 0.05). The circular data markers indicate estimated hazard ratios. The error bars represent the 95% CIs. **(B)** Kaplan–Meier curves for the high- (n = 27) and low- (n = 37) risk score patient groups in the TCGA-STAD Asian cohort. Log-rank test, p = 0.001. **(C)** Kaplan–Meier curves for the high- (n = 150) and low- (n = 150) risk score patient groups in the TCGA-STAD Asian cohort. Log-rank test, p = 0.023. **(D)** Kaplan–Meier curves for the high (n = 108) and low (n = 98) risk score patient groups in the TCGA-STAD Asian cohort. Log-rank test, p = 0.222. **(E)** TGFB1 expression in gastric cancer. Local patients were dichotomized into high- and low-risk subgroups at the cutoff point (8.68) of the histochemistry score of TGFB1 expression. **(F)** Kaplan–Meier curves for the high- (n = 20) and low- (n = 108) risk score patient groups in the local patients. Log-rank test, p < 0.001.

To further analyze ASIRGPI-related molecular targets, 29 immunomodulatory genes that could be detected in the entire cohort of 364 patients were explored ; only two (EDNRB and TGFB1) were found to be positively correlated with the ASIRGPI (r ≥ 0.40, P < 0.05) and significantly associated with poor outcomes (**Figure 7A**). OS analysis (**Figures 7B, C**) further proved that expression of the therapeutic target TGFβ1 was only significantly associated with poor outcome of the AGC patients (TCGA-STAD Asian cohort and ACRG cohorts), while no such phenomenon was found in the TCGA-STAD White cohort (**Figure 7D**).

To verify the effect of TGFB1 on Asian gastric cancer, IHC detection of TGFB1 was performed in the TMA. The TGFB1 expression in 35 pairs of matched tissues was analyzed (**Supplementary Figure S4A**). The TGFB1 expression was significantly higher in the tumor tissue (p = 0.031, **Supplementary Figure S4B**). Among all samples, 83.8% (129 of 154) were TGFB1-positive overall. A cutoff point of 8.68, which was optimized using a ROC, was chosen to categorize patients into TGFB1 high and low expressed subgroups. Further survival analysis found that the high-TGFB1-expressed patients had a shorter OS than the low-expressed patients (p < 0.001; **Figures 7E, F**).

## Discussion

GC patients often display heterogeneous clinical outcomes, with OS ranging from months to decades (2, 8). Even among patients at the same TNM stage and receiving the same treatment, survival outcomes vary widely (15). In this regard, treatments are limited, as all GC patients receive a similar therapeutic regimen, lacking individual differences. Considering the striking differences in both the incidence rate and OS of the disease between Asian and Western countries (19), we first paid special attention to this difference and identified gastric cancer patients by ethnicity. Interestingly, we found that the activation of TGFβ signaling as well as TGF-β1-induced EMT was linked with the poor prognosis of white GC patients compared with that of the Asian cohort. This offers new insights for accurately identifying high-risk patients and novel therapeutic targets for AGC patients.

Recently, several studies have focused on survival-associated IRG expression profiles and developed individualized cancer prognostic signatures (20–23). Zhang et al. confirmed that the IRGs and TIICs, which are indispensable components of the tumor microenvironment, are significantly associated with survival outcomes in GC (24). Thus, we first attempted to analyze the prognostic value of IRGs and TIICs in Asian patients with GC. In this study, 35 differentially expressed IRGs in AGC that are significantly associated with OS were identified using public AGC cohorts. LASSO Cox regression analysis was performed, and seven IRGs were identified to be significantly associated with AGC progression. We developed a robust ASIRGPI model based on the seven IRGs and proved its efficacy in the ACRG cohort. Additionally, the unavailability of our ASIRGPI model for Caucasians further confirmed its Asian specificity. The correlations of overall survival with age, gender, TNM stage, and ASIRGPI were analyzed, and the ASIRGPI was determined as an independent predictor for outcomes, which could provide potential practical guidance for individual therapeutic regimens and improved antitumor immune responses in AGC.

Previous studies (24–26) have shown that TIICs are highly associated with tumorigenesis, invasion, and metastasis. The interactions between TIICs and tumor cells are considered to be directly associated with physical tumor cell destruction, tumor burden reduction, and clinical prognosis improvement. In this study, we also examined the relationship between TME subcomponents and ASIRGPI and outcomes in AGC patients. Considering that our ASIRGPI was based on IRGs, we found that the ASIRGPI was strongly and positively related to the stromal and immune scores. However, in spite of this correlation, only stromal score was relevant to the prognosis of AGC patients, which is consistent with the results of a previous study (27). This shows that the abundance of stromal components is independently related to the prognosis of GC. Hence, we infer that the effect of ASIRGPI on the worse survival of AGC patients may be related to the remodeling of stromal components; moreover, further TME cell type analysis also revealed that stromal cells accounted for the largest proportion of the nine cell types that were positively correlated with both prognosis and ASIRGPI. In summary, our results provide new insight into the mechanism by which ASIRGPI regulates the prognosis of AGC patients.

To further explore potential ASIRGPI-based therapeutic targets for AGC patients with poor prognosis, a panimmune gene set was performed and the TGFβ-related gene set was revealed to be significantly correlated with ASIRGPI and poor survival. Similar to previous studies, which demonstrated that high expression levels of TGFβ1 were associated with poor prognosis in several cancers as well as GC (27, 28), further immunomodulatory gene analysis also indicated that TGFβ1 are significantly correlated with ASIRGPI and poor survival. Our experimental verification in GC tissue microarrays also confirmed this conclusion at the protein level. Additionally, since TGF-β signaling pathway activation is regarded as a symbol of extracellular matrix dysregulation and EMT (29), we also found that TGFB1 was highly expressed in extracellular matrices in the GC tissue microarrays. On this basis, we infer that the poor prognosis in both white GC patients and AGC patients with high ASIRGPI can be attributed to the remodeling of stromal components *via* TGF-β signaling pathway activation; however, the exact mechanism requires further study. It is worth mentioning that TGFβ1 was only found to be significantly associated with poor outcome of the AGC patients. Galunisertib and M7824, molecules targeted to block the TGFβ signaling pathway, have already been used in the clinical treatment of a variety of cancers (30–32); they may now become a new treatment for AGC rather than for all GC patients.

Despite our significant findings, this study had certain limitations. First, although a well-validated prognostic model was established, we only focused on AGC patients; the sample size was relatively small, and further validation of local data is necessary. Second, we gathered TCGA-STAD and ACRG/GSE62254 cohorts; sampling bias in sequencing methods on account of the differences in platforms used is inevitable. Finally, our study provides new insight into the AGC stromal microenvironment and related potential targets for individual therapy. However, this was a retrospective study; prospective studies and in-depth investigations into their functions are urgently needed to confirm these findings.

In summary, this study is the first to systematically demonstrate that AGC patients with good prognosis have lower TGFβ signaling enrichment. We further analyzed the role of IRGs in monitoring the survival of AGC and developed and validated a survival-associated IRG-based ASIRGPI model. This may have important clinical implications for the survival outcomes of AGC. Finally, we comprehensively analyzed the TME characterization to explore related survival mechanisms based on ASIRGPI and found that TGFB1 may be a suitable novel target for individual AGC therapy.

## Data Availability Statement

The datasets presented in this study can be found in online repositories. The names of the repository/repositories and accession number(s) can be found in the article/**Supplementary Material**.

## Ethics Statement

The studies involving human participants were reviewed and approved by the local Research Ethics Board of The Wenzhou Medical University Second Affiliated Hospital. The patients/participants provided their written informed consent to participate in this study.

## Author Contributions

XY and XS conceived and designed the study. CM, LM, YH, and XY were responsible for data acquisition. HH and WC have verified the underlying data. CM, LM, YH, XY, and AS were responsible for analysis of data. CM, LM, and RG were responsible for the interpretation of data. CM, XX, and XS prepared the manuscript with assistance from all authors. All authors were responsible for the revision of the manuscript, approval of the final version for publication, and accuracy and integrity of the work.

## Funding

Contract grant sponsor: the National Natural Science Foundation of China, contract grant numbers 81672707 (to XS) and 31670922 (to XX); contract grant sponsor: the Wenzhou Basic Scientific Research Projects, contract grant number Y20180064 (to CM).

## Conflict of Interest

The authors declare that the research was conducted in the absence of any commercial or financial relationships that could be construed as a potential conflict of interest.

## Publisher’s Note

All claims expressed in this article are solely those of the authors and do not necessarily represent those of their affiliated organizations, or those of the publisher, the editors and the reviewers. Any product that may be evaluated in this article, or claim that may be made by its manufacturer, is not guaranteed or endorsed by the publisher.
