# Supplementary figures and images for: Immunogenomic Landscape and Immune-Related Gene-Based Prognostic Signature in Asian Gastric Cancer
Source: Front Oncol. 2021 Nov 5;11:750768. doi: 10.3389/fonc.2021.750768 (PMC8602354; doi:10.3389/fonc.2021.750768)

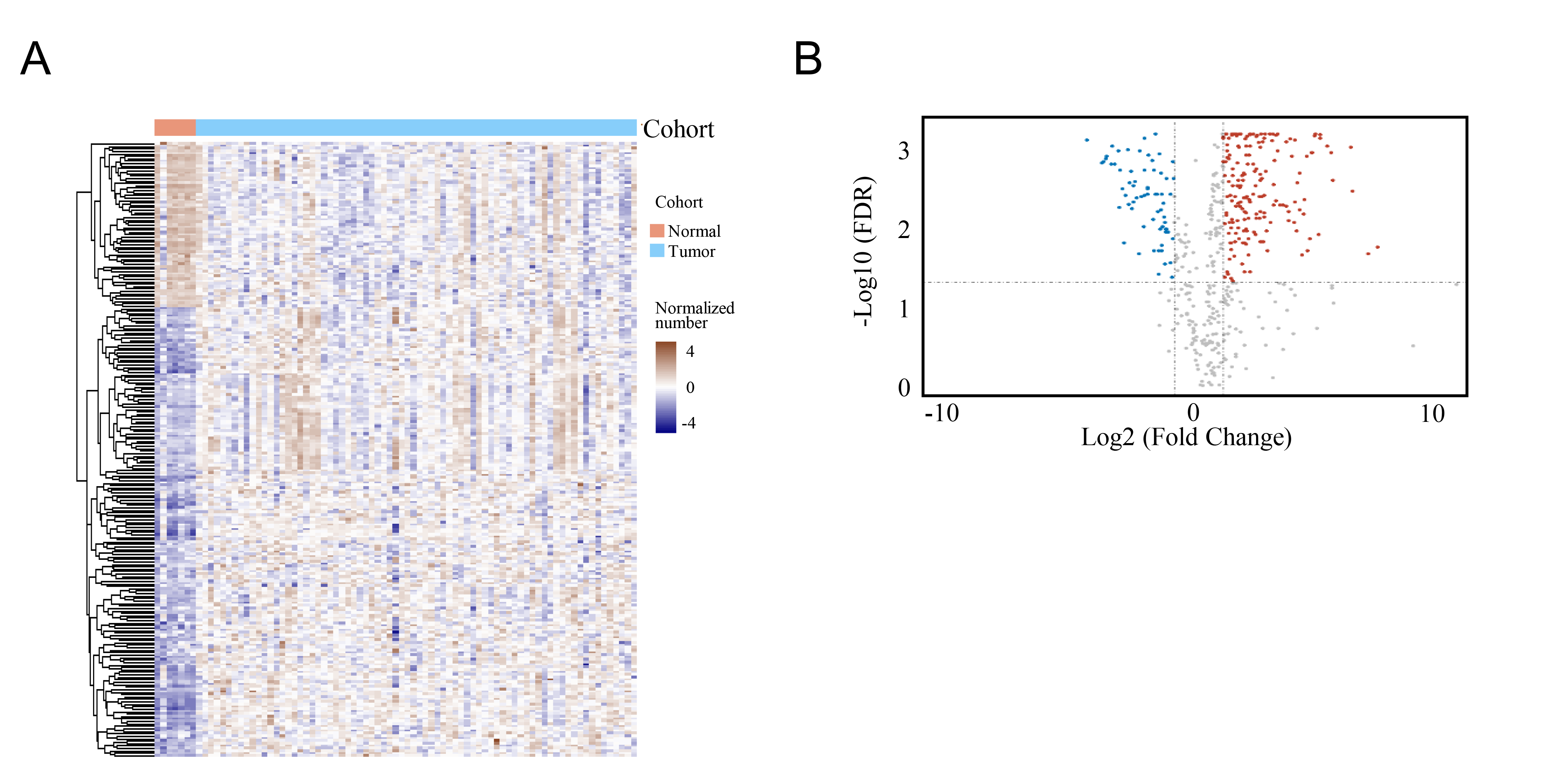

Supplement: Supplementary file 1 [file Image_1.tif]

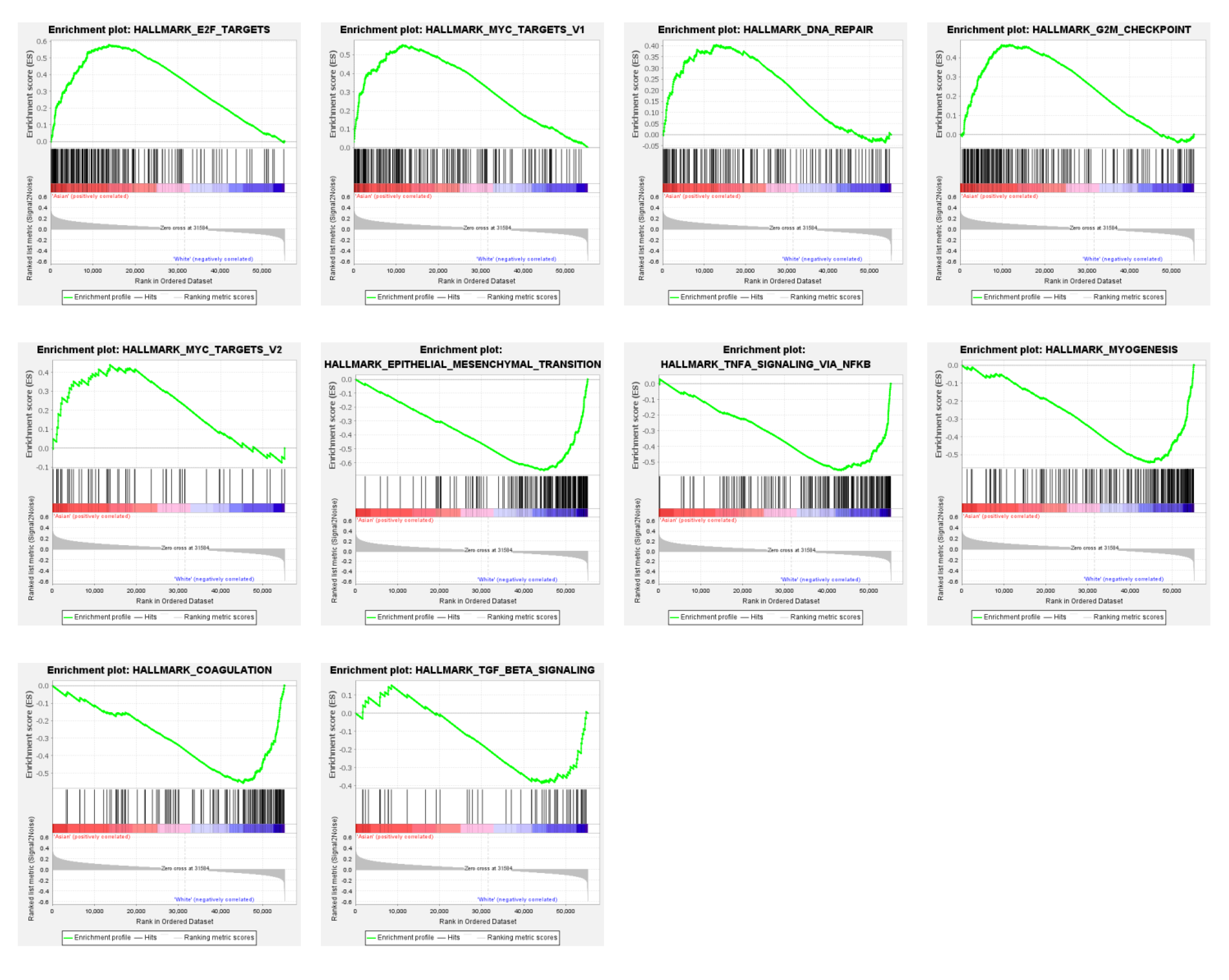

Supplement: Supplementary file 2 [file Image_2.tif]

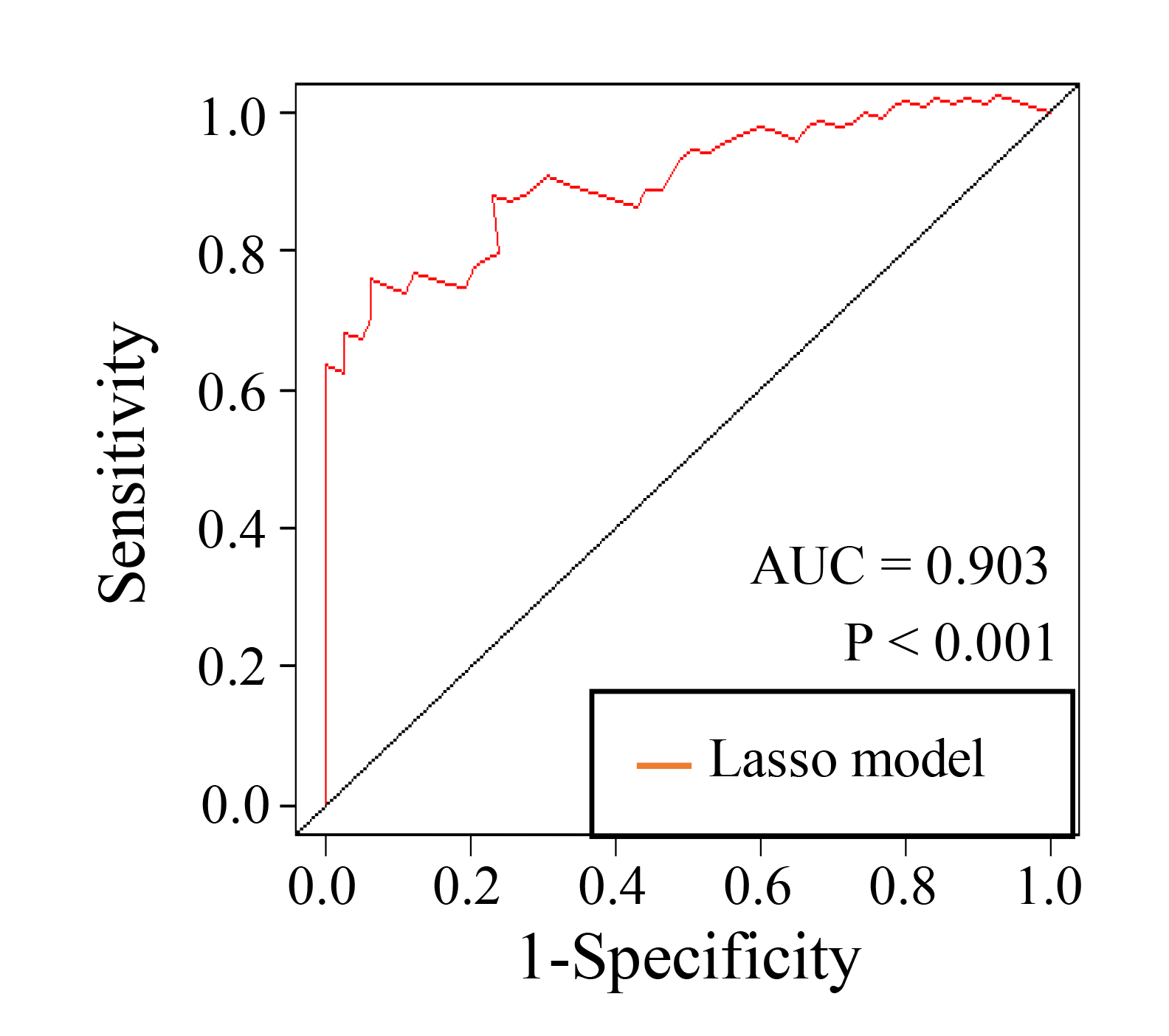

Supplement: Supplementary file 3 [file Image_3.tif]

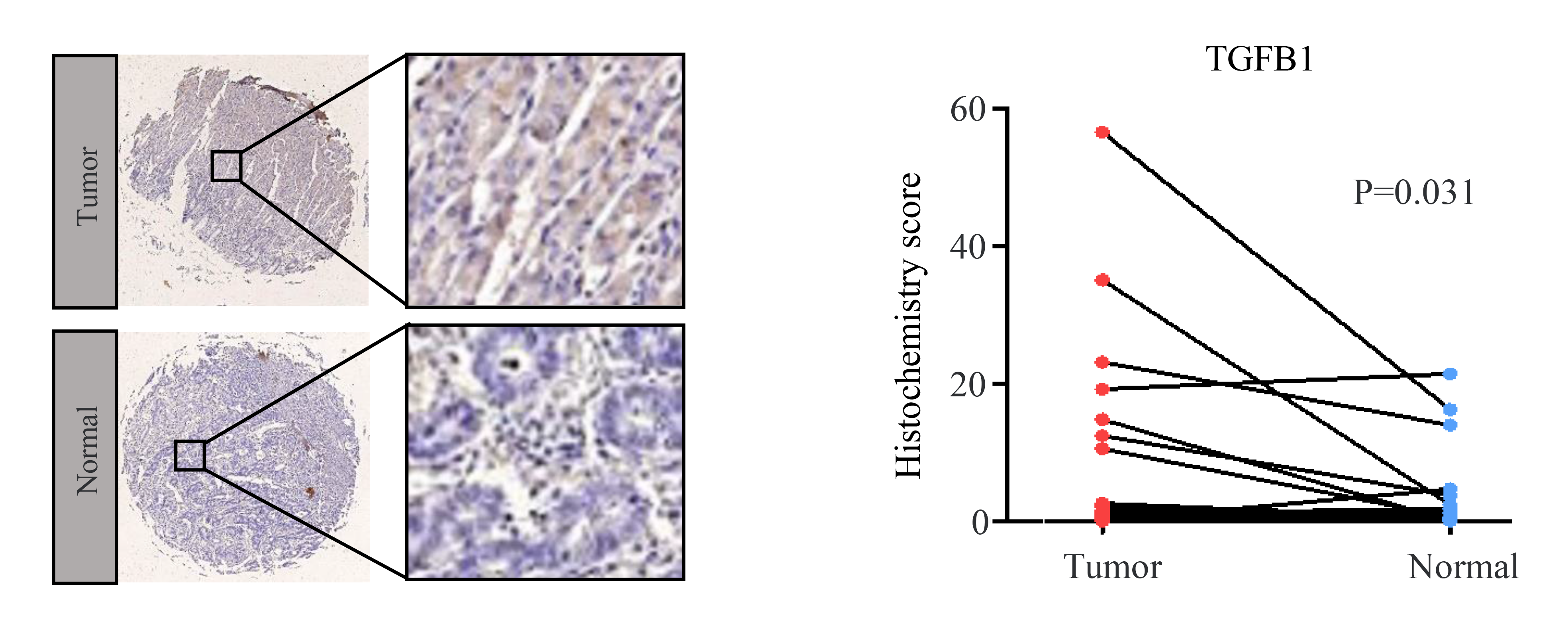

Supplement: Supplementary file 4 [file Image_4.tif]
